# Epidural cerebellar stimulation drives widespread neural synchrony in the intact and stroke perilesional cortex

**DOI:** 10.1186/s12984-021-00881-9

**Published:** 2021-05-26

**Authors:** Aamir Abbasi, Nathan P. Danielsen, Jennifer Leung, A. K. M. G. Muhammad, Saahil Patel, Tanuj Gulati

**Affiliations:** 1 Center for Neural Science and Medicine, Departments of Biomedical Sciences and Neurology, Cedars-Sinai Medical Center, Los Angeles, CA USA; 2 PhD Program in Biomedical Sciences, Graduate School of Biomedical Sciences, Cedars-Sinai Medical Center, Los Angeles, CA USA; 3 Department of Medicine, David Geffen School of Medicine, University of California-Los Angeles, Los Angeles, CA USA; 4 Department of Bioengineering, Henri Samueli School of Engineering, University of California-Los Angeles, Los Angeles, CA USA

**Keywords:** Motor cortex, Cerebellum, Epidural direct current stimulation, Neural plasticity

## Abstract

**Background:**

Cerebellar electrical stimulation has shown promise in improving motor recovery post-stroke in both rodent and human studies. Past studies have used motor evoked potentials (MEPs) to evaluate how cerebellar stimulation modulates ongoing activity in the cortex, but the underlying mechanisms are incompletely understood. Here we used invasive electrophysiological recordings from the intact and stroke-injured rodent primary motor cortex (M1) to assess how epidural cerebellar stimulation modulates neural dynamics at the level of single neurons as well as at the level of mesoscale dynamics.

**Methods:**

We recorded single unit spiking and local field potentials (LFPs) in both the intact and acutely stroke-injured M1 contralateral to the stimulated cerebellum in adult Long-Evans rats under anesthesia. We analyzed changes in the firing rates of single units, the extent of synchronous spiking and power spectral density (PSD) changes in LFPs during and post-stimulation.

**Results:**

Our results show that post-stimulation, the firing rates of a majority of M1 neurons changed significantly with respect to their baseline rates. These firing rate changes were diverse in character, as the firing rate of some neurons increased while others decreased. Additionally, these changes started to set in during stimulation. Furthermore, cross-correlation analysis showed a significant increase in coincident firing amongst neuronal pairs. Interestingly, this increase in synchrony was unrelated to the direction of firing rate change. We also found that neuronal ensembles derived through principal component analysis were more active post-stimulation. Lastly, these changes occurred without a significant change in the overall spectral power of LFPs post-stimulation.

**Conclusions:**

Our results show that cerebellar stimulation caused significant, long-lasting changes in the activity patterns of M1 neurons by altering firing rates, boosting neural synchrony and increasing neuronal assemblies’ activation strength. Our study provides evidence that cerebellar stimulation can directly modulate cortical dynamics. Since these results are present in the perilesional cortex, our data might also help explain the facilitatory effects of cerebellar stimulation post-stroke.

## Background

The cerebellum and* M1* are heavily interconnected brain areas that play a vital role in motor control and learning [1, 2]. Different classes of cerebellar neurons have been linked to different movement features. For example, deep cerebellar nuclei (*DCN*) neurons, the principal projection neurons from the cerebellum to *M1*, are tuned to cues leading to movement onset and duration in reaching tasks [3–10]. Purkinje cells, another principal cell type in the cerebellar cortex, are correlated to limb position, velocity, distance of limb movement and muscle activity during movement. Moreover, Purkinje cell activity becomes more synchronized during the learning of a skilled reaching task [3, 4, 11–18]. In addition, studies have revealed a strong link between *M1* neural plasticity and motor skill acquisition [19–22]. Consistent with these findings, other recent work has shown that inactivation of either area leads to a loss of kinematic precision in skilled reaching tasks [10, 23].

Studies have also shown that cerebellar stimulation can modulate motor function, motor excitability and cerebellar plasticity [24–28]. This work, along with accumulating evidence that cerebellar stimulation can influence remote functional connectivity, has generated interest in assessing the role of cerebellar stimulation as a potential therapy for movement disorders, and in stroke rehabilitation [29–33]. Animal studies have stimulated *DCN* neurons in rodent stroke models. These results demonstrated that subjects showed subsequent improvements in motor behavior, and changes in MEPs, axonal growth protein and synaptogenetic markers in peri-infarct cortices [34–36]. Other animal work has shown that epidural stimulation of the cerebellum has a smoothing effect on corticomotor maps [24], and that cerebellar transcranial electric stimulation (tES) can entrain neurons in the cerebellar cortex and exerts its effects principally through modulation of Purkinje cells [37].

While these studies have provided key insights into the effects of cerebellar stimulation, our current understanding of how cerebellar stimulation impacts ongoing dynamics in the cortex at the level of single neurons or neural ensembles remains limited. Furthermore, how this stimulation modulates ongoing activity in the peri-infarct cortex can help in understanding the facilitatory effects of cerebellar stimulation during stroke rehabilitation. Analyzing single neuronal activity at the cortical level can provide useful details that may aid in optimizing cerebellar stimulation paradigms. We were also interested in assessing coordinated firing of neural ensembles after cerebellar stimulation, as neuronal co-firing in a temporally coupled manner is known to be important for information processing and plasticity in the brain [38–42]. It is likely that such coordinated activity may play an important role in driving neural plasticity after injury and during neuromodulatory approaches such as cerebellar stimulation [34, 35].

In this study, we have developed a model to study the effects of cerebellar stimulation on cortical activity using acute large-scale extracellular recordings of intact *M1* and stroke-injured peri-infarct *M1*. We were particularly interested in understanding the diversity of single neuron responses to epidural cerebellar stimulation because epidural stimulation is less invasive than deep stimulation, and therefore presents a lower translational barrier [42, 43]. It is unlikely that all neurons respond to cerebellar stimulation in the same manner due to differences in cell type, and the diversity of single neuron network connectivity [44]. We were also interested in assessing changes in neural synchrony, and examined the effects of cerebellar stimulation on *M1* single neuron firing rates, as well as *M1* ensemble dynamics like coordinated firing and neuronal pair coupling. We found that cerebellar stimulation could significantly change both the firing rate and the synchronous firing of neurons in the intact and stroke-injured perilesional *M1*. Together our results provide evidence that epidural cerebellar stimulation directly modulates neural dynamics in the intact and the peri-infarct *M1*.

## Methods

### Animal preparation

Adult male Long-Evans rats were used in this study (*n* = 9, 250–400 g, ~ 8 weeks old, Charles River Laboratories). All animal procedures were performed according to the protocols approved by the Institutional Animal Care and Use Committee at Cedars-Sinai Medical Center, Los Angeles. This ensured that the animals that were used in this research were acquired, cared for, housed, used, and disposed of in compliance with the applicable federal, state and local laws and regulations, institutional policies and with international conventions to which the United States is a party. Animals were housed on a 14 h light and 10 h dark cycle (Photoperiod is from 6 am to 8 pm) in a climate controlled vivarium. One animal was excluded from the study due to significant recording drift and electrical noise in the recording. Thus *n* = 8 animals were used for the analysis. Out of these, four rats were assigned to the intact group and another 4 to the acute stroke group. Before starting the stimulation surgery, animals were briefly anesthetized using isoflurane, and then injected with a ketamine/xylazine cocktail (85 mg/kg and 10 mg/kg, respectively). Supplemental ketamine (42.5 mg/kg) was given every 30–50 min as needed to maintain the anesthesia level. The ketamine–xylazine anesthetic state is predominantly characterized by slow wave oscillations in the neocortex [45], and has been used in other studies to investigate the effects of electric stimulation on ongoing cortical activity dominated by low-frequency oscillations [42, 46]. In addition to anesthesia, atropine (0.05 mg/kg) was administered to counter respiratory or cardiac depression. Animals were perfused and their brains collected for staining on the same day after recordings.

### Stimulation

**Fig. 1:**
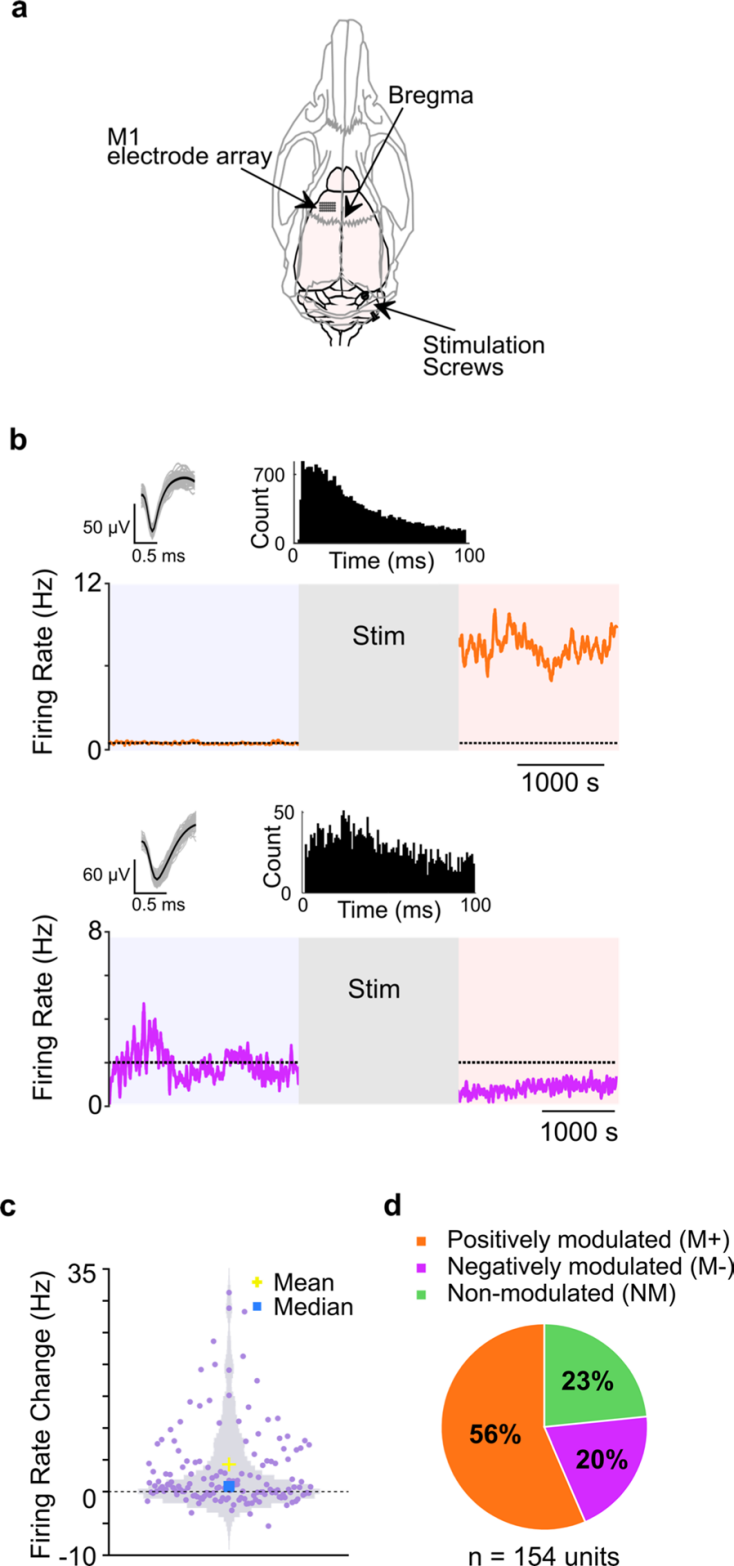
Diversity in firing rate response of *M1* units after cerebellar stimulation. **a** Direct current stimulation was applied to the cerebellum while neural activity of contralateral *M1* was recorded using an electrode array. **b** Example of either a significant increase (orange) or decrease (purple) in firing rate after cerebellar stimulation (p < 0.05). Top panel shows waveforms and the distribution of inter-spike interval of respective units (100 representative waveforms are plotted for each unit and the inter-spike interval is from full recording period). The dotted lines represent the mean from *Stim*_*pre*_. **c** Violin plot showing the change in firing rate from *Stim*_*pre*_ to *Stim*_*post*_ across all neurons. **d** Percentage of positively (*M*^+^) or negatively (*M*^−^), and non-modulated (*NM*) units across all animals

After anesthetizing the animal, cranial screws were implanted in the skull in the configuration noted in Fig. 1a to act as epidural cerebellar stimulation electrodes. The stimulation screws were implanted over the cerebellar hemisphere contralateral to recorded *M1*. The location of the first screw was 11.5 mm posterior and 2.5 mm lateral to bregma. The second screw was placed 14 mm posterior and 3.0 mm lateral to bregma. This arrangement mimics a rostrocaudally oriented electric field, which has been shown to result in the greatest amount of Purkinje cell modulation [37, 47]. Our stimulation electrode montage roughly encompassed the posterior lobe of the cerebellum; the anterior bone screw was connected to the cathode (current magnitude: − 175 μA), and the posterior screw served as the ground (Fig. 1a). A direct current stimulation (DCS) was applied for 30 min through these electrodes. DCS was applied directly onto the dura to ensure a defined contact area over the cerebellar cortex. Our current density was estimated at 0.25 mA/mm^2^ based on our previous work [42]. This is comparable to that used in invasive human and non-human primate studies [48, 49].

### Electrophysiological recordings

We recorded extracellular neural activity using 32 channel tungsten microwire electrode arrays (MEAs, Tucker-Davis Technologies (Alachua, FL): each 33-μm polyimide-coated tungsten electrode). These were 8 × 4 arrays with 250 μm spacing in between each electrode in a row and 375 μm spacing between rows (total 4 rows). The long axis of the probe was placed mediolaterally in* M1*. Following a craniotomy and a durectomy over the forelimb area of *M1*, arrays were lowered down to 900–1500 μm from the surface of the dura mater. In the intact animals, the recording array was centered at 0.5–1 mm anterior to bregma and 2.5–3.5 mm lateral from midline, and in stroke rats, it was placed slightly anterior to the stroke site, centered at 3–3.5 mm anterior to bregma and 2.5–3.5 mm lateral to midline. Depth was finalized based on quality of recordings across the array. A ZIF-clip-based digital head stage with a TDT-RZ2 system was used for signal acquisition. We recorded spikes at 24,414 Hz and LFPs at 1017 Hz sampling frequencies. The average duration of recordings was 124.84 ± 8.31 min (mean ± s.e.m.) across all animals, where pre-stimulation baseline activity (*Stim*_*pre*_) was recorded for 43.54 ± 4.46 min and post-stimulation activity (*Stim*_*post*_) was recorded for 43.57 ± 4.43 min. We used Plexon offline sorter (Plexon, Dallas, TX) to perform post-hoc spike sorting. A total of 348 single units were isolated across eight animals.

### Photothrombotic stroke

After the craniotomy, rose bengal dye (20 mg/kg) was injected into the femoral vein over a 2 min period using an intravenous catheter. Next, the *M1* area of the brain was illuminated with a green laser (532 nm, Laserglow Technologies) through a 2-mm aperture (centered 0.5 mm anterior and 2.5 mm lateral to bregma) for 12 min, while the remaining exposed cortex was covered with a custom aluminum foil mask to prevent light penetration. After induction, a probe was implanted in the perilesional cortex immediately anterior to the stroke site [42, 50]. The craniotomy or implanted electrodes were covered with a layer of silicone (Kwik Sil™).

### Data analysis

#### Single unit analyses

After spike sorting, further single unit analyses were performed in MATLAB (Rb2018a) using a combination of custom written routines. We started by selecting high amplitude units for subsequent analyses. In order to identify units with high amplitude, we calculated the signal-to-noise ratio (SNR) for every recorded unit using the following equation [50, 51]:

$$SNR = \frac{A}{{2*SD_{noise} }}$$

where *A* is the peak-to-peak voltage of the mean spike waveform and *SD*_*noise*_ is the standard deviation of the baseline noise floor. Our single unit analysis was typically done on units with SNR > 3.5. For during-stimulation analyses in intact animals, we chose neurons with SNR > 6.

#### Firing rate analysis

Firing rate was calculated from the spike trains of isolated single units by counting the number of spikes in the 50 ms bins. Using this method, we calculated the mean firing rate in the *Stim*_*pre*_* Stim*_*dur*_ and *Stim*_*post*_ periods. Firing rates were calculated for the entire duration of all periods. A significant change in firing was estimated by calculating the mean post-stimulation firing rate and checking if it was outside of the 95% distribution of pre-stimulation firing rate. Neurons that increased their firing rate were classified as positively modulated (*M*^+^), while ones that decreased their firing rate were classified as negatively modulated (*M*^−^). The rest of the units were classified as non-modulated (*NM*) units.

#### Spike train cross-correlation

We began by equaling the spike counts in pre- and post-stimulation periods. We then computed cross-correlation histograms (CCH) using 10 ms bins for all neuronal pairs. Furthermore, we constructed pseudo-random spike train CCHs with simulated spike counts of equal length for every pairing in *Stim*_*pre*_ and *Stim*_*post*_ conditions. Such simulations were run 300 times (Monte Carlo Simulations) [52]. CCH counts were converted to probability. Thereafter, to quantify pairwise coincident firing from *Stim*_*pre*_ to *Stim*_*post*_, we took the mean of the simulated CCH probability within ± 400 ms around the center and subtracted it from the mean of real CCH probability in the same window for *Stim*_*pre*_ and *Stim*_*post*_ conditions, respectively (*Δ*_*CCH*_).

#### Ensemble activation analysis

Next, we wanted to assess changes in the activity patterns of neural ensembles. For this, we characterized the neural activity patterns in *Stim*_*pre*_ and *Stim*_*post*_ by comparing it to a template that was created by performing principal component analyses (PCA) on a baseline *Stim*_*pre*_ neural activity matrix. This method can detect cell assembles or neurons with shared activity patterns [40, 52–54]. To perform these analyses, we computed a pairwise unit activity correlation matrix from baseline activity during *Stim*_*pre*_. *Stim*_*pre*_ spike trains were binned (*t*_bin_ = 500 ms) for each neuron. These spike trains were z-transformed, and then organized into a 2-D matrix of neurons (rows) by time (columns). From this spike count matrix, we calculated the correlation matrix and then calculated the eigenvector for the largest eigenvalue from this correlation matrix and used it as the ensemble baseline activity template. We then equaled the number of spikes in *Stim*_*pre*_ and *Stim*_*post*_, and then projected the ensemble baseline activity template back onto them. This projection is a linear combination of Z-scored binned neural activity from the two blocks above, weighted by the principal component (PC) ensemble (i.e., the eigenvector) that was calculated from the baseline activity matrix. This linear combination has been described as the “activation strength” of that particular ensemble. Typically, the significance of a PC is determined by *λ*_*max*_, which is the highest eigenvalue that arises out of an equivalently sized random matrix based on the Marchenko–Pastur law. We got 1–3 significant PCs from the baseline activity, but the first PC explained the highest variance of the data so we focused our analysis on that one. Using this method, we compared the activation strength of the first PC in each animal during *Stim*_*pre*_ and *Stim*_*post*_. *Stim*_*dur*_ time periods were excluded from ensemble analyses as we restricted during-stimulation analyses to a limited number of very high SNR neurons, and ensemble analyses are more well-suited to datasets with a larger number of neurons.

#### LFP power analysis

LFP analyses were conducted using a combination of custom-written routines in Matlab, along with functions from the Chronux toolbox (http://chronux.org/) [55]. Pre-processing for LFP analyses involved the elimination of periods with artifacts and the removal of broken channels and noisy segments of LFPs by offline visual inspection. Additionally, ± 10 s of LFP data around the start and end of stimulation was removed to reduce segments with artifacts. We calculated power on a total of 32 channels across eight animals. The electrodes we implanted in *M1* were 32-channel arrays that were arranged as eight shanks in four rows (375 μm row separation). We selected one channel from each row for these analyses, totaling four channels from each animal. Power analyses were performed by time matching the *Stim*_*pre*_ and *Stim*_*post*_ time periods or *Stim*_*pre*_ and *Stim*_*dur*_ time periods to the shortest recording period, splitting them into 10 s segments and then finding the mean power across the segments. We used a time-bandwidth product of 10 with 19 tapers for multitaper spectral analyses [46]. Mean power was calculated across the *δ-*band (0.3–4 Hz, which is the predominant oscillation with ketamine anesthesia), and all the values in this frequency range were averaged together on each channel. Statistical analyses were performed on the average power estimates of this frequency band respective to *Stim*_*pre*_*, Stim*_*dur*_ and *Stim*_*post*_ values (see section below).

### Staining for stroke

After the acquisition of brain electrophysiological recordings was complete, the rats were re-anesthetized with isoflurane inside a desiccator jar and intracardially perfused with phosphate buffered saline (PBS) for 10 min followed by 4% paraformaldehyde in PBS for 30 min. The brains were harvested and post-fixed in 4% paraformaldehyde in PBS for 4 h at the end of which they were transferred to 30% sucrose (w/v) in 0.1 M PBS until equilibrated. The tissue was embedded in Tissue-Tek O.C.T. compound, cryosectioned into 50 µm sagittal sections and stored in 0.1% Sodium Azide in PBS at 4 °C. For Fluoro-Jade C staining, the free-floating sections were given three washes with PBS followed by mounting and air drying at room temperature. The slides were then incubated with gentle shaking in the following solutions in sequence: 1% sodium hydroxide in 80% ethanol for 5 min, 70% ethanol for 2 min, distilled water (DW) for 2 min, 0.06% potassium permanganate for 15 min, and DW for 2 min. The slides were protected from light during the subsequent steps: incubation with freshly prepared 0.0001% Fluoro-Jade C (Chemicon International, CA) in 0.1% acetic acid with DAPI for 30 min, 3 washes with DW (2 min each time), air dry for 5 min, and placement on slide warmer at 65 °C for 10 min. Finally, the dried slides were cleared in Xylenes and cover-slipped using DPX mounting medium. Digital images of the stained sections were collected on an Olympus BX51W1.

### Statistical analysis

**Fig. 2:**
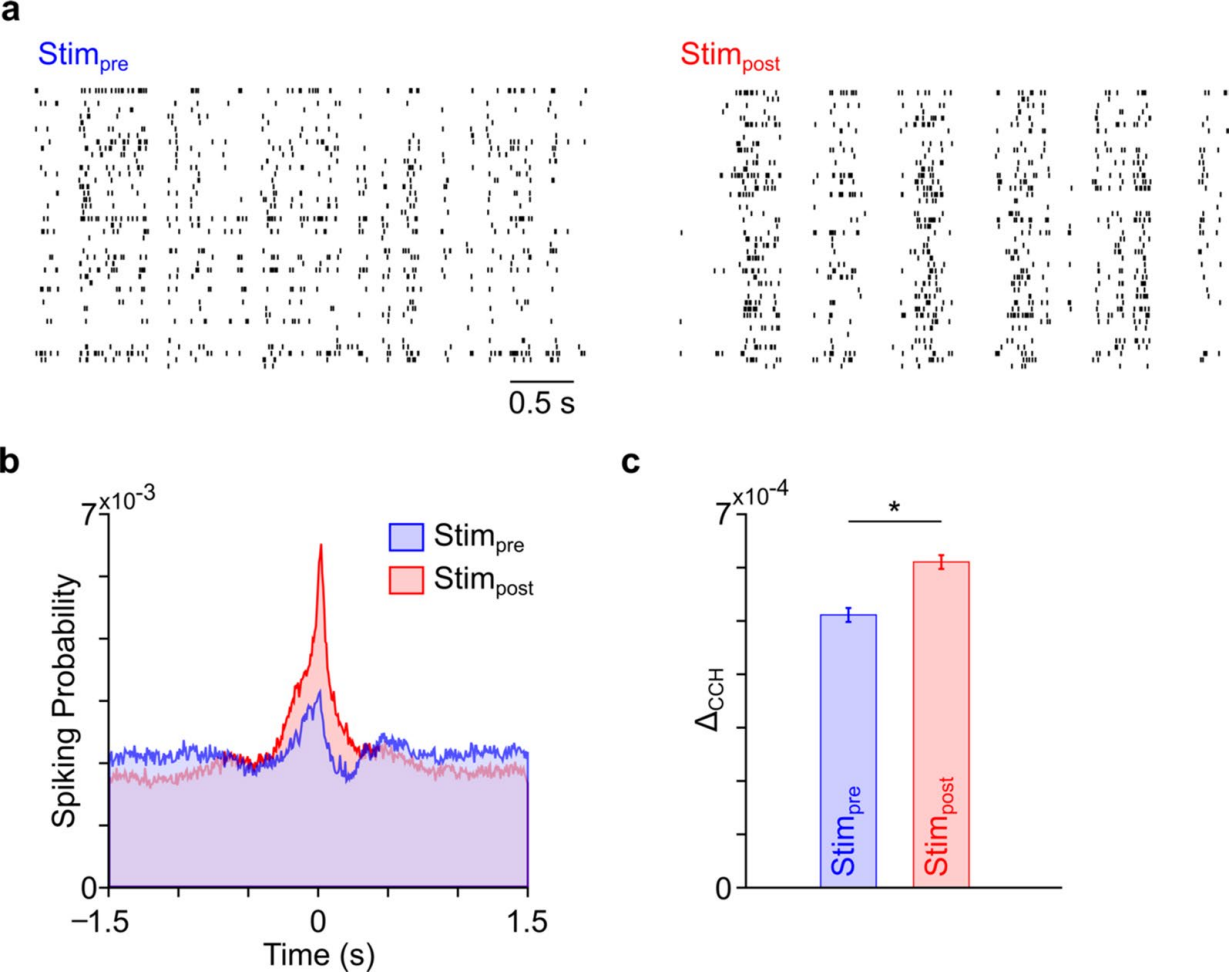
*M1* coincident firing is strengthened post cerebellar stimulation. **a** Raster plots depicting changes in correlated activity among *M1* units from *Stim*_*pre*_ to *Stim*_*post*_. **b** Representative cross-correlogram of a pair of *M1* units, before and after cerebellar stimulation. **c** Mean changes in *Δ*_*CCH*_ are shown as mean ± s.e.m. across four animals. *p < 1 × 10^–67^

**Fig. 3:**
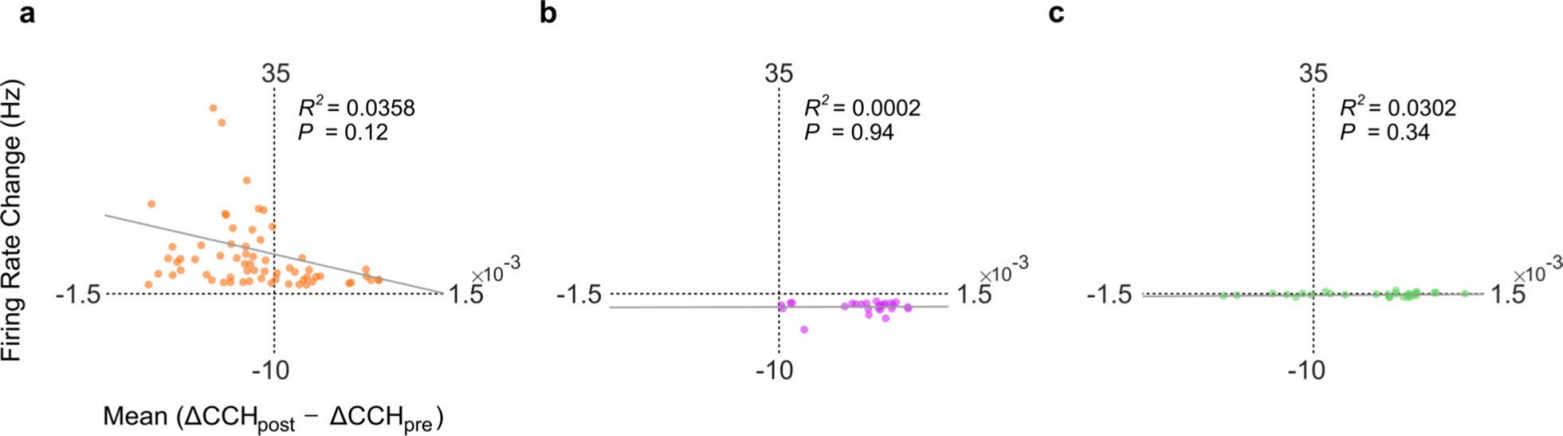
Changes in neural synchrony are unrelated to firing rate changes. Regression analysis of the mean *ΔCCH* change from *Stim*_*pre*_ to *Stim*_*post*_ for a neuron with all other neurons with similar modulation in a rat (labeled *Mean* (ΔCCH_post_ − ΔCCH_pre_)) compared to the same neuron’s firing rate change from *Stim*_*pre*_ to *Stim*_*post*_ is shown. **a** Regression for *M*^+^ neurons firing rate change and correlation change. **b** Similar regression for *M*^−^* neurons* and **c** for *NM* neurons are shown

**Fig. 4:**
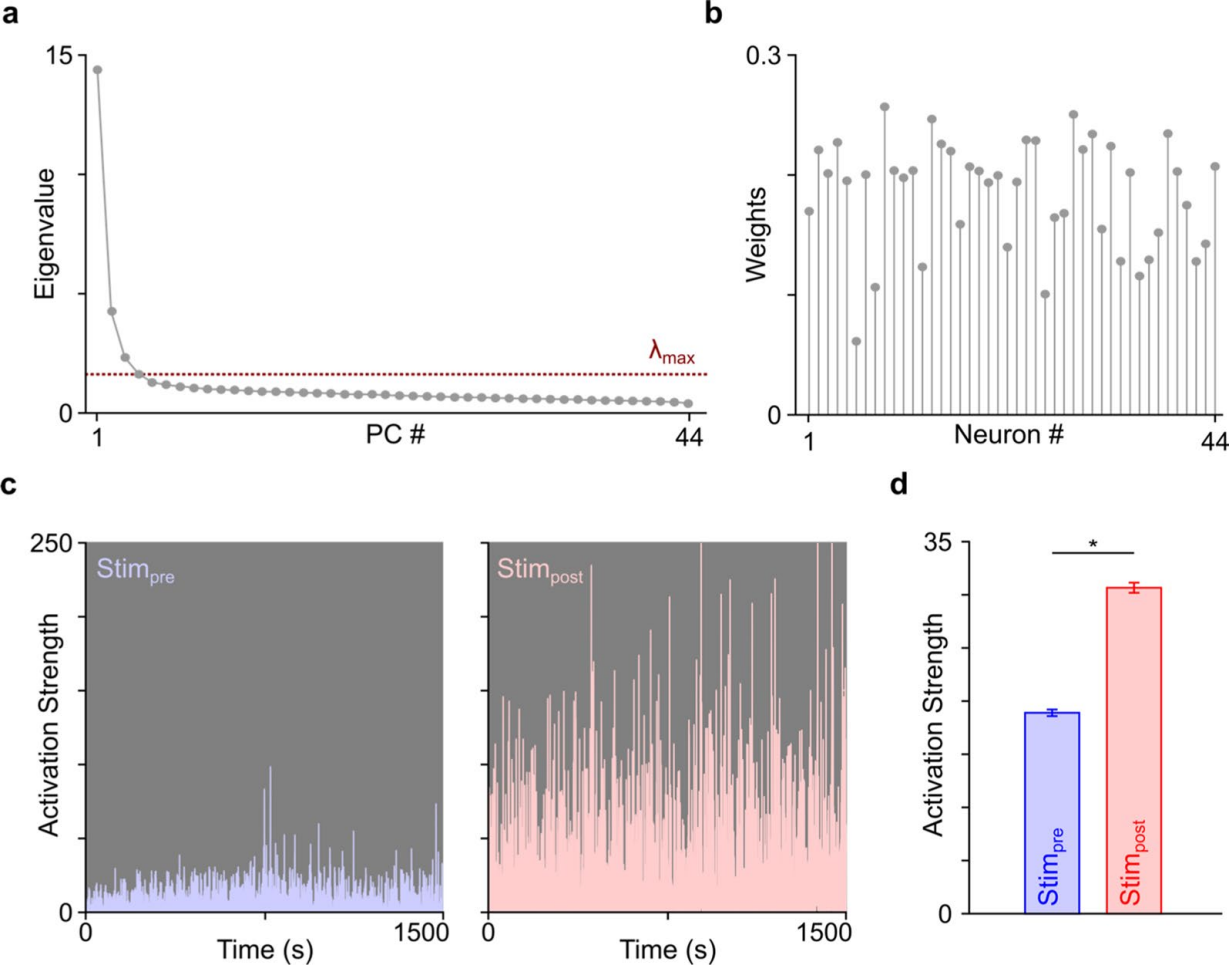
Activation of *M1* ensembles before and after cerebellar stimulation. **a** Correlation matrix eigenvalues calculated from spontaneous activity prior to cerebellar stimulation. The dashed line is the signal threshold (*λ*_*max*_), defined as the theoretical upper bound for a randomized spike train. Three PCs have eigenvalues greater than *λ*_*max*_. **b** The weight of each neuron contributing to the first principal component (or ensemble) in **a**. **c** Example of activation events of an *M1* ensemble prior to and after cerebellar stimulation (i.e., *Stim*_*pre*_ and *Stim*_*post*_). **d** Across all animals, there was a significantly stronger activation of *M1* ensembles post stimulation (average of top 20th percentile activation events in *Stim*_*pre*_ and *Stim*_*post*_ blocks, *p < 10^–115^)

**Fig. 5:**
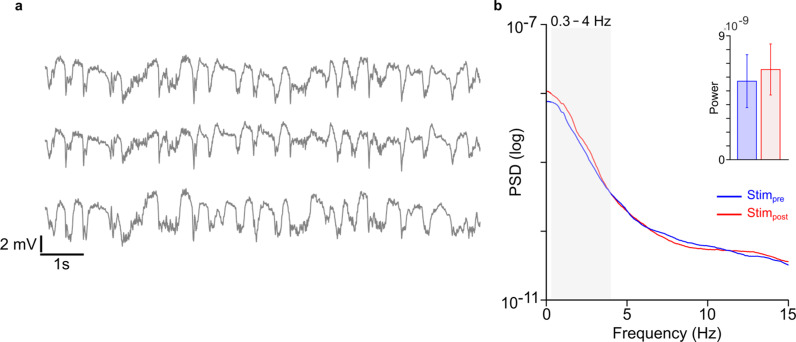
Power spectrum analyses of *M1* LFP. **a** An example of LFP traces from three channels in an animal. **b** An example LFP power from a single animal, before and after cerebellar stimulation. Grey shaded area shows the slow frequency band (0.3–4 Hz). *Inset,* the average LFP power in the slow frequency band across animals. No significant changes were observed in this frequency band (*p* = 0.69)

**Fig. 6:**
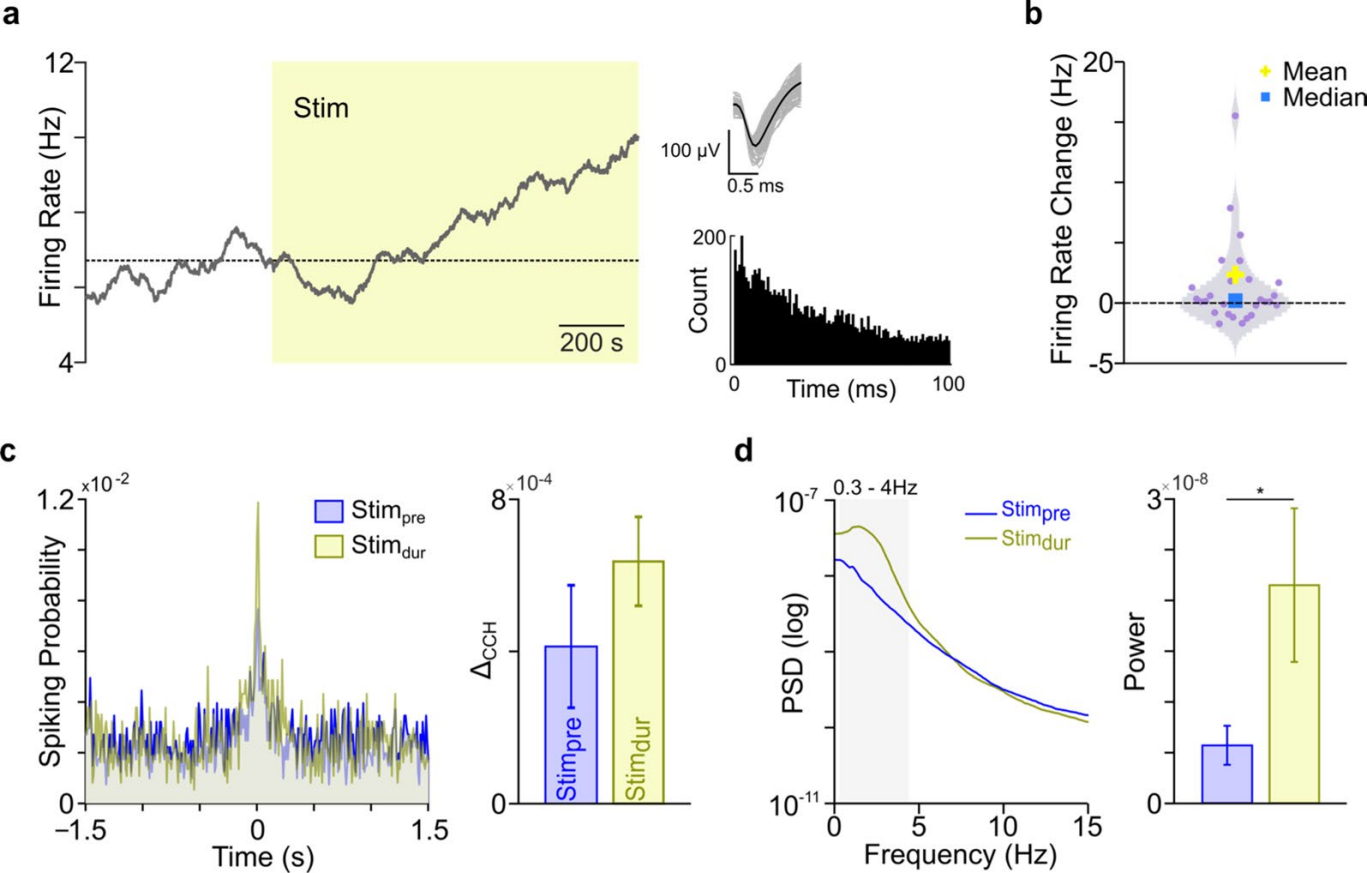
*M1* activity during epidural cerebellar stimulation. **a** An example *M1* unit showing modulation during stimulation (left). Its waveform and inter-spike interval histogram is shown (right). **b** Violin plot showing firing rate change between *Stim*_*pre*_ and *Stim*_*dur*_ across all high-amplitude *M1* units. **c** Representative cross-correlogram of a pair of *M1* units, before and during cerebellar stimulation (left). Mean change in the *Δ*_*CCH*_ is shown as mean ± s.e.m., across 4 animals (right). **d **Change in LFP power from a single animal before and during cerebellar stimulation (left). Grey shaded area shows the slow frequency band (0.3–4 Hz). On the right is the average LFP power in the slow frequency band across all animals (right, *p < 1 × 10^–2^; *Stim*_*pre*_ power is same in Fig. 5 but y-axis scale is adjusted to accommodate *Stim*_*dur*_ power)

**Fig. 7:**
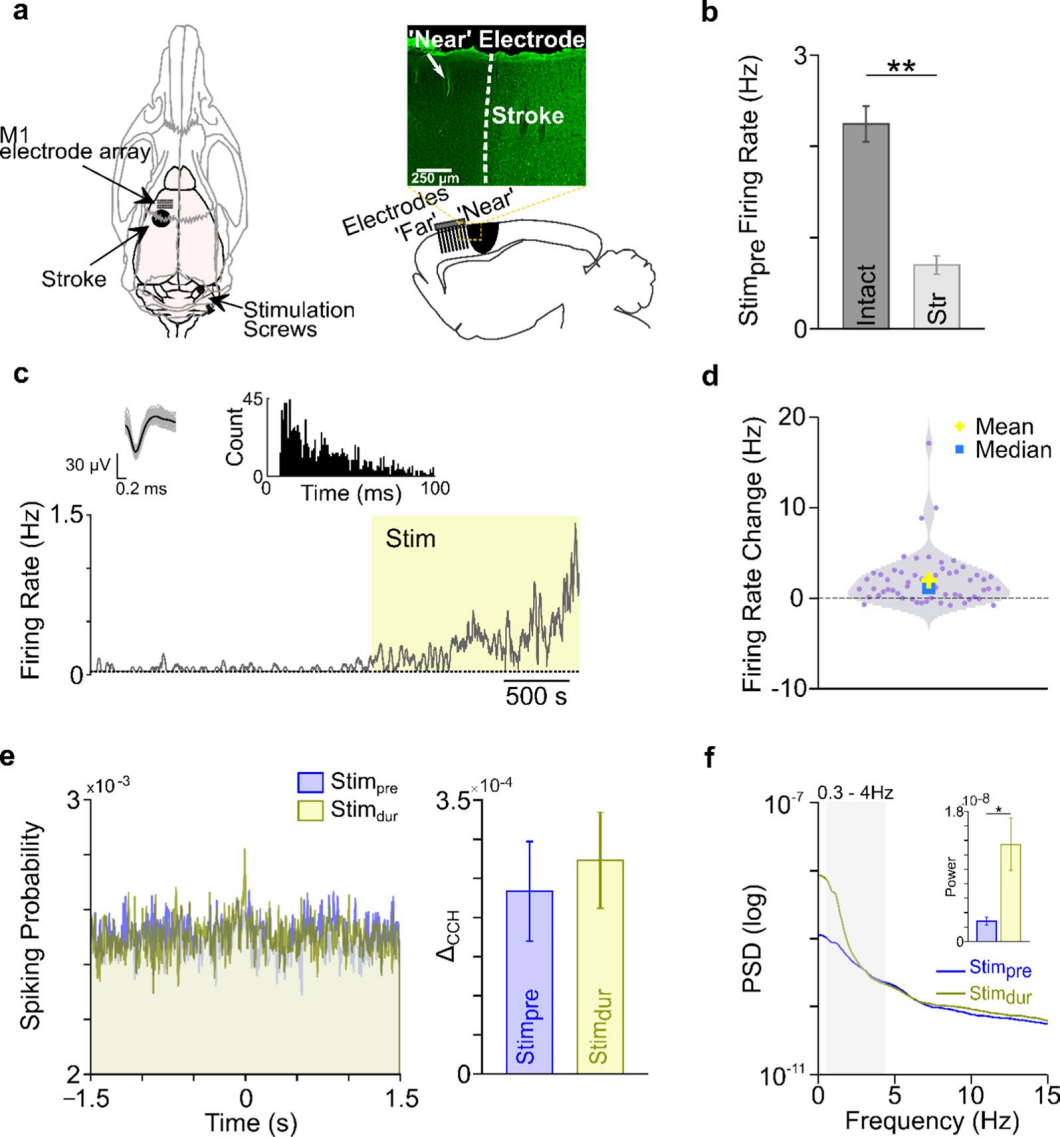
Activity in post-stroke peri-infarct *M1* during cerebellar stimulation. **a** A schematic of electrophysiological recordings in stroke peri-infarct *M1* and cerebellar stimulation (left). A histology image with Fluoro-Jade C staining showing the sagittal view of stroke area and the site of electrophysiologcal recordings. **b** Mean *Stim*_*pre*_ firing rate of intact and stroke animals is shown as mean ± s.e.m. (Str: stroke; **p < 5 × 10^-8^). **c** An example peri-infarct *M1* unit showing modulation during stimulation (bottom). Its waveform and inter-spike interval histogram is also shown. **d** Violin plot showing firing rate change between *Stim*_*pre*_ and *Stim*_*dur*_ across all high-SNR peri-infarct *M1* units. **e** Representative cross-correlogram of a pair of peri-infarct* M1* units, before and during cerebellar stimulation (left). Mean change in *Δ*_*CCH*_ is shown as mean ± s.e.m., across four animals (right). **f **An example LFP power from a single animal before and during cerebellar stimulation (left). Grey shaded area shows the slow frequency band (0.3–4 Hz). The average LFP power in the slow frequency band across four animals is shown on the right side, *p < 1 × 10^–2^

**Fig. 8:**
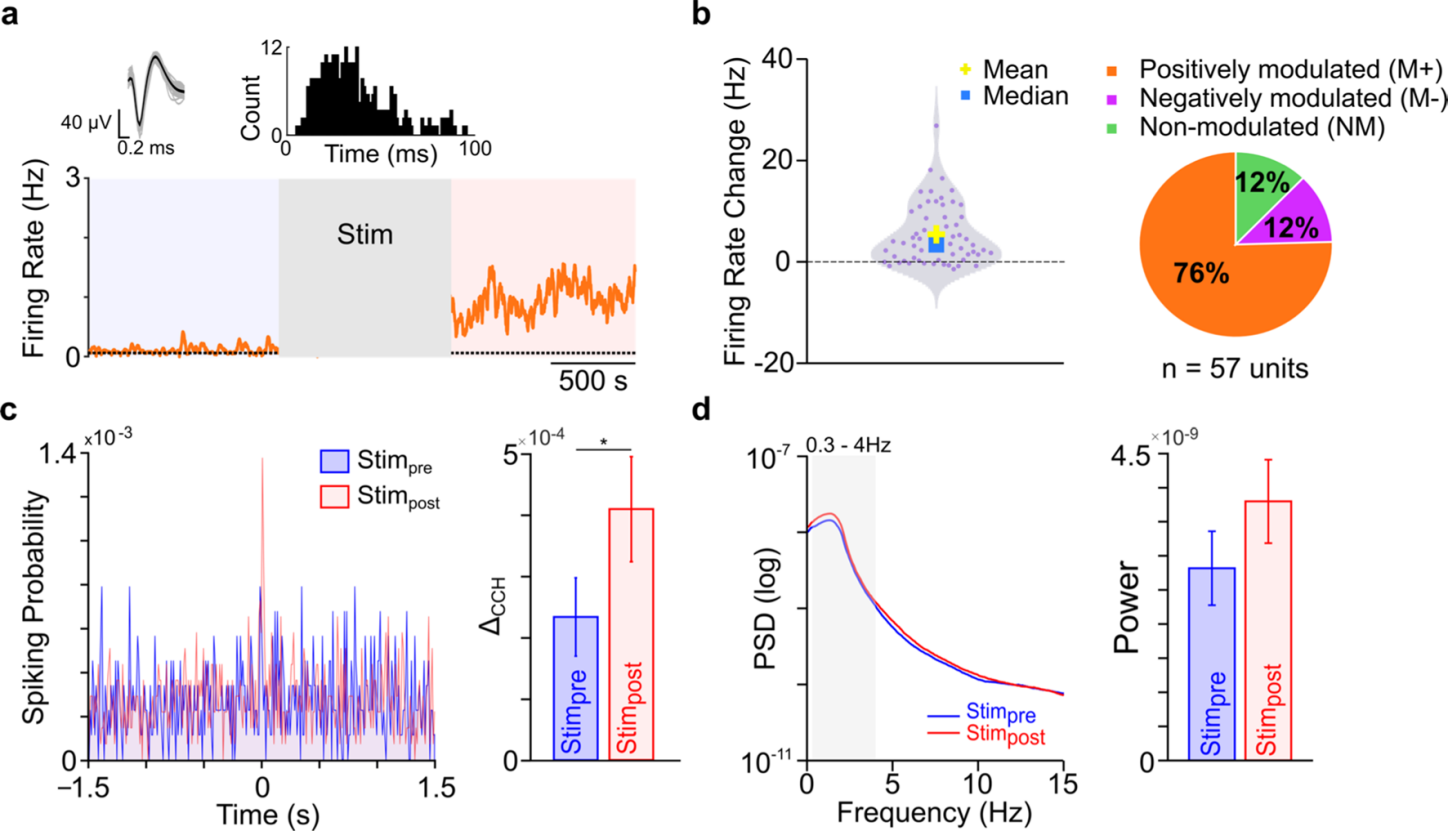
Activity in post-stroke peri-infarct *M1* after cerebellar stimulation. **a** An example peri-infarct *M1* unit showing modulation after cerebellar stimulation (bottom). Its waveform and inter-spike interval histogram is also shown (top). **b** Violin plot showing firing rate change between *Stim*_*pre*_ and *Stim*_*post*_ across all peri-infarct *M1* units (left) and percentage of *M*^+^, *M*^−^ and *NM* units across all stroke animals (right). **c** Representative cross-correlogram of a pair of peri-infarct *M1* units, before and during cerebellar stimulation (left). Mean change in *Δ*_*CCH*_ is shown as mean ± s.e.m., across four animals (right). *p < 5 × 10^–2^. **d** An example LFP power from a single animal before and after cerebellar stimulation (left). Grey shaded area shows the slow frequency band (0.3–4 Hz). The average LFP power in the slow frequency band across four animals is shown in the inset (*Stim*_*pre*_ power is same as the Fig. 7 but y-axis is adjusted)

In this study, we performed statistical analyses by implementing routines in MATLAB (Rb2018a). The linear mixed-effects model (implemented using MATLAB *fitlme*) was used to compare the differences between *Stim*_*pre*_ and *Stim*_*post*_ groups shown in Figs. 1c, 2c, 4d, 5b, 6b–d, 7b, d, e, f and 8b–d. This model accounts for the ‘nested’ datasets wherein multiple observations have been collected from the same research subject [42, 56]. In Fig. 3, we computed the differences in firing rates of all individual neurons from *Stim*_*pre*_* to Stim*_*post*_ and correlated them with the difference between the mean ΔCCH change of the respective neuron with all other neurons from *Stim*_*pre*_ to *Stim*_*post*_. We used linear regression (implemented using MATLAB *fitlm*) to evaluate changes in firing rate and coincident firing changes after cerebellar stimulation for pairs of neurons showing an increase (Fig. 3a), decrease (Fig. 3b) or no change in firing rate from *Stim*_*pre*_ to *Stim*_*post*_ (Fig. 3c).

## Results

### Cerebellar stimulation modulates neural firing in intact M1

We first analyzed the effects of cerebellar stimulation on the firing rate of *M1* neurons in the intact cortex (Fig. 1). The majority of recorded neurons showed a significant change in their firing rate following stimulation. Examples of both a significantly positively modulated (*M*^+^, *Stim*_*pre*_ firing rate = 0.56 ± 0.01 Hz, *Stim*_*post*_ firing rate = 7.40 ± 0.02 Hz, p < 0.05) and a significantly negatively modulated (*M*^*−*^, *Stim*_*pre*_ firing rate = 1.97 ± 0.01 Hz, *Stim*_*post*_ firing rate = 0.848 ± 0.007 Hz, p < 0.05) neuron are shown in Fig. 1b. Additionally, we observed a significant change in the firing rate of neurons at the population level (*n* = 154 neurons, mixed-effect model, *p* < 10^–7^, see Fig. 1c), and found that at the individual neuron level 56% of neurons showed increased firing, 20% showed decreased firing and 23% showed no significant change in firing in *Stim*_*post*_ (Fig. 1d).

To ensure that fluctuations in baseline firing rate were not due to anesthesia, we analyzed firing rate changes from early and late *Stim*_*pre*_, and found that they did not change significantly (*n* = 154 neurons, baseline change = 0.037 ± 0.01 Hz, mixed-effect model, *p* > 0.05). Additionally, we found no significant change in baseline LFP power in the *δ-*band within *Stim*_*pre*_ (mixed-effect model, *p* > 0.05).

### Cerebellar stimulation increases neural synchrony

To analyze changes in neural synchrony post cerebellar stimulation in the intact brain, we looked at how the magnitude of cross-correlation between pairs of *M1* units changed from *Stim*_*pre*_ to *Stim*_*post*_ (Fig. 2a). We analyzed a total of 2425 neuron pairs for this analysis. An example of an increase in cross-correlation from *Stim*_*pre*_ to *Stim*_*post*_ is shown in Fig. 2b. At the population level, we calculated the magnitude of the difference between the peaks of *Stim*_*pre*_ and *Stim*_*post*_ from a shuffled correlogram (*Δ*_*CCH*_, see “Methods”). We found a significant increase in correlated firing post-stimulation (mean *Stim*_*pre*_ to *Stim*_*post*_ change 29.51%; mixed-effect model, *p* < 10^–67^, see Fig. 2c). Thus, even though firing rates were diversely modulated following cerebellar stimulation, neural activity became consistently more synchronized.

### Neural synchrony is independent of changes in M1 firing rate

As shown above, cerebellar stimulation significantly modulated neural firing rates and correlated firing between neurons. While we equaled the number of spikes in *Stim*_*pre*_ and *Stim*_*post*_ before making cross-correlograms (see “Methods”), it is possible that our observed changes in coincident firing were related to the changes in the firing rate. We therefore examined whether there was a relationship between the firing rate changes of a neuron and the change in *Δ*_*CCH*_ of that neuron with other neurons. We specifically wanted to check if neurons that increased their firing rate (i.e., *M*^+^ neurons) also experienced an increase in neural synchrony. Similarly, we also wanted to check if the neurons that decreased their firing rates (*M*^−^ neurons) experienced a reduction in their cross-correlograms. Hence, we focused on the correlations changes of neurons that were part of either *M*^+^*–M*^+^ or *M*^−^*–M*^−^ pairs. We also performed another regression for neurons of *NM–NM* pairs and their respective cross-correlations to compare to *M*^+^/*M*^−^ neurons. We found that only 3.5% of the variation in change of *Δ*_*CCH*_ was explained by firing rate changes of *M*^+^ neurons (Fig. 3a), 0.02% was explained by *M*^−^ neurons (Fig. 3b) and 3.02% was explained by NM neurons (Fig. 3c). These results indicate that changes in correlated firing were not correlated with the firing rate changes of positively modulated, negatively modulated or non-modulated neuron pairs. This suggests that the effects of cerebellar stimulation on *M1* neural synchrony are independent of its effects on the firing rates.

### Activation strengths of M1 cell assemblies is strengthened after epidural cerebellar stimulation

Having demonstrated large-scale cerebellar stimulation-dependent changes in firing rate and correlated firing among neural pairs, we next examined whether cerebellar stimulation boosted the activation of *M1* cell assemblies over their baseline level of activation (*Stim*_*pre*_) in the intact brain. To investigate this, we used principal component analyses (PCA) to identify patterns of neural activity (i.e., neural ensembles) and then probed their activation magnitude before and after stimulation (see “Methods” for details).

Ensemble activation during the *Stim*_*pre*_ and *Stim*_*post*_ periods was quantified by applying a PCA-generated template of spontaneous baseline neural activity from pre-stimulation recordings. PCA resulted in a number of principal components (PCs or “ensembles”) that reflected patterns of common variance across the recorded single-units, with each component comprised of weights that reflected the contribution of each neuron to that particular ensemble (Fig. 4a, b). To represent the activity of a particular ensemble, the traditional method is to multiply the weights from each neuron in the ensemble with the z-scored activity matrix of the recorded neurons. The ensemble defined from the spontaneous pre-stimulation recording period was multiplied by the z-scored neural activity recorded during the *Stim*_*pre*_ and *Stim*_*post*_, resulting in a one-dimensional vector that represents the “activity” of that ensemble before and after stimulation (Fig. 4c).

After stimulation, ensemble activation strength was significantly stronger compared to pre stimulation (top 20th percentile activation strengths, activation strength in *Stim*_*pre*_ = 18.90 ± 0.31 and *Stim*_*post*_ = 30.66 ± 0.47; mixed-effect model, *p* < 10^–115^, Fig. 4d). These results indicate that epidural cerebellar stimulation strongly boosted neural ensemble activation.

### M1 LFP power remains unchanged after cerebellar stimulation

Next, we examined the mesoscopic changes in *M1* activity by looking at LFP signals. The ketamine–xylazine anesthetic state is predominantly characterized by slow wave oscillations in the neocortex as depicted in Fig. 5a [45]. These slow oscillations are similar in frequency to recently described low-frequency oscillations (LFOs) that can serve as a biomarker for stroke recovery [42], and hence have been studied to assess the effects of electric stimulation [42, 46]. We analyzed power changes in low-frequency bands (i.e., δ-band, 0.3–4 Hz). Our LFP analysis revealed no significant change in δ-band LFP power post cerebellar stimulation (14.82%, mixed-effect model, *p* = 0.69, Fig. 5b).

### Changes in M1 activity start to set-in during cerebellar stimulation

Whether the above-described changes in *M1* activity started to set in with the onset of stimulation requires an analysis of during-stimulation changes (*Stim*_*dur*_). Due to increased noise levels during cerebellar stimulation, we considered only neurons with very high amplitudes (SNR > 6) for this analysis. We found that the changes in firing rate of positively (*M*^+^) and negatively modulated (*M*^−^) neurons start to set in during stimulation. An example of an *M*^+^ neuron is shown in Fig. 6a. The distribution of changes in the firing rates of units before and during cerebellar stimulation across *M*^+^ and *M*^−^ groups is shown in Fig. 6b. These units show significant changes in their firing rates during stimulation (mixed-effect model, *p* < 0.05). We also observed that coincident firing starts to increase during stimulation (Fig. 6c), but this increase was not significant (n = 59 pairs, mixed-effect model, *p* = 0.36). Furthermore, we looked at δ-band LFP power during stimulation. We found a significant increase in power from *Stim*_*pre*_ to *Stim*_*dur*_ (276.12%, mixed-effect model, *p* < 0.05, Fig. 6d). These changes in power spectrum show that low-frequency oscillatory activity in *M1* is boosted during cerebellar stimulation. However, the power spectral changes were not long-lasting and ended by *Stim*_*post*_, while the firing rate changes and coincident firing changes persisted. Together, these results indicate that the effects of cerebellar stimulation on single units persist long after stimulation has ceased, and that they start to set in during stimulation.

### Changes in peri-infarct M1 during cerebellar stimulation

In our next set of experiments we wanted to check for concordance of the results we observed in healthy animals with acutely stroke-injured animals. We induced a photothrombotic stroke in *M1* in four animals and recorded activity directly anterior to the site of stroke in peri-infarct *M1* before, during and after cerebellar stimulation (Fig. 7a). Since there was a paucity of neurons that we could record after stroke, our analyses were done on all neurons with SNR greater than 3.5 (n = 57). We first looked at the firing rate of units in intact and stroke brain. We found that firing rates of units in the stroke perilesional cortex were lower than the firing rates of units in the intact *M1* during *Stim*_*pre*_ (Fig. 7b). Thereafter, we looked at changes in the firing rates of peri-infarct *M1* neurons during cerebellar stimulation. An example of an *M*^+^ neuron is shown in Fig. 7c. The units from stroke perilesional cortex showed significant changes in their firing rates during stimulation (Fig. 7d, mixed-effect model, *p* < 10^–3^). We also looked at changes in the coincident firing of peri-infarct *M1* units. Although coincident firing starts to increase during stimulation, this increase was not significant (Fig. 7e, mixed-effect model, *p* = 0.60). Next, we looked at δ-band LFP power during stimulation, and found a significant increase in power from *Stim*_*pre*_ to *Stim*_*dur*_ (376.70%, mixed-effect model, *p* < 0.01, Fig. 7f). Overall, recordings from stroke-injured rats show similar trends to recordings in intact rats during cerebellar stimulation.

### Changes in peri-infarct M1 post cerebellar stimulation

We also looked at the *Stim*_*post*_ changes in peri-infarct *M1* activity. The firing rate changes that start to set in during stimulation persist after stimulation as well. An example of an *M*^+^ neuron with a sustained increase in spiking activity is shown in Fig. 8a. At the population level, units from peri-infarct *M1* showed significant changes in their firing rates after stimulation (mixed-effect model, *p* < 10^–7^) and 88% of cells changed their firing rate significantly (Fig. 8b). Furthermore, we observed a significant change in the coincident firing of peri-infarct *M1* units post cerebellar stimulation (Fig. 8c, mixed-effect model, *p* < 0.05). Our LFP analysis from *Stim*_*post*_ in stroke-injured rats showed a nonsignificant increase in δ-band LFP power (34.73%; mixed-effect model, *p* = 0.66, Fig. 8d).

## Discussion

Our work shows that epidural cerebellar stimulation can significantly change neural firing rates and induce *M1* plasticity that lasts 30–45 min after the end of stimulation. Moreover, neural synchrony as measured through coincident firing changes (*Δ*_*CCH*_) increased significantly irrespective of the direction of the change in firing rate after stimulation. These changes occurred over a mesoscale oscillatory backdrop of low-frequency oscillations due to ketamine–xylazine anesthesia, and we observed these changes in the intact and stroke-injured *M1*. These results suggest that cerebellar stimulation can directly modulate *M1* dynamics and increase cortical neural synchrony. Our study helps support other recent findings that report the facilitatory effects of cerebellar stimulation in stroke recovery [34–36, 57, 58].

### Relation to previous models of cerebellar stimulation

Previous studies have shown that cerebellar stimulation can alter functional connectivity and excitability in cortical areas [24, 26, 27]. These changes have been evaluated using functional imaging [26], which is an indirect measure of neural activity. Moreover, cerebellar stimulation has been shown to increase cortical excitability in rodent and human studies as evaluated through MEPs induced either through transcranial magnetic stimulation (TMS) or intracortical microstimulation (ICMS) [27, 36]. Both deep brain stimulation (DBS) and transcranial direct current stimulation (tDCS) have been studied extensively for their applications in abnormal motor behavior. Our work can be likened to tDCS studies as we stimulated the cerebellum epidurally. Cerebellar tDCS is shown to induce polarity specific modulation in the cerebellar cortex [59]. This work suggested that anodal tDCS excites the cerebellar cortex, whereas cathodal tDCS exerts an inhibitory effect. Furthermore, computational modeling work has shown that tDCS directly affects cerebellar circuitry, and that stimulation currents are contained within the cerebellum [60]. An index of cerebellar brain inhibition (CBI) has been used to evaluate the impact of cerebellar modulation [61], and it was noted that cathodal tDCS reduces CBI and anodal tDCS increases CBI (although another study found that anodal tDCS reduces CBI [62]). These observed effects have been explained either through a direct effect of tDCS on inhibitory neurons in the cerebellar cortex or on cerebello-thalamo projections to inhibitory interneurons of *M1*. Epidural cerebellar stimulation in animal models supports these findings, where it has been shown that epidural cerebellar stimulation via anodal current injection focuses corticomotor maps by augmenting inhibition. Meanwhile, cathodal epidural stimulation has the reverse effect [24]. New work recently showed that epidural cerebellar stimulation principally affects the main inhibitory cell in the cerebellar cortex, the Purkinje cell [37]. The cerebellar cortex strongly inhibits the cerebellar nuclei. Hence, inhibiting the cerebellar cortex can result in excitatory overdrive along the dentato-thalamo-cortical (DTC) pathway [63]. This work is indicating that there are polarity-dependent site-specific effects of direct current stimulation in the cerebellar cortex, similar to that which has been reported in cerebral cortex [64, 65]. Our work is in agreement with these findings, as we placed a cathode over cerebellar posterior lobes and largely observed an increase in contralateral *M1* neurons’ firing rates.

Furthermore, DBS of the DTC pathway in rodents has also shown very promising results, extending the early studies that showed that single pulse stimulation of the dentate nucleus modulated cerebral cortical excitability [66–68]. This novel DBS approach used a continuous stimulation that targeted the DTC pathway, and reported that low-frequency beta-band stimulation enhanced cortical excitability and promoted functional recovery [34, 69]. This work also showed that DBS helped reduce crossed cerebellar diaschisis [70, 71]. Our work is also consistent with the facilitatory effects of lateral cerebellar nuclei (LCN) stimulation in these studies, as we find that cerebellar stimulation induces a heightened neural synchrony in the contralateral peri-infarct *M1* that is amenable to plasticity.

Our work adds to this body of literature and shows how *M1* activity is directly affected by cerebellar stimulation. Specifically, our work has demonstrated three main points: First, epidural cerebellar stimulation can directly modulate the firing patterns of *M1*. This is demonstrated by the changes in firing rates of single neurons. Second, the diverse changes in neural firing rate we found suggest a more complex neural response to cerebellar stimulation. A better understanding of the diversity of responses and their neural bases, possibly through the study of their underlying connectivity, might help improve the efficacy of cerebellar stimulation. Third, our results suggest that cerebellar stimulation may act by changing spontaneous firing rate or neural synchrony.

### Cerebellar stimulation and neural plasticity

Cerebellar stimulation induced plasticity appears to affect recorded *M1* neurons differently. While most neurons experienced a change in firing rate, the extent and the direction of change was variable. We can envision several possible mechanisms for this diversity. Recent work that stimulated the cerebellum using transcranial alternating current showed that multiunit activity of the cerebellar cortex was enhanced during the negative phase of stimulation, while the positive phase suppressed activity [37, 47]. This work also showed that Purkinje cells were the main cell type that was affected by transcranial epidural cerebellar stimulation [37]. Similarly, anodal DCS over the cerebellum has been shown to have a ‘smoothing’ effect on corticomotor maps [24]. Finally, theta burst stimulation has been shown to induce cerebellar plasticity [24]. The cerebellar cortex sculpts its output to M1 by adjusting the firing rate and timing of the neurons in the *DCN*. It is thus likely that our observations of changes in neural synchrony in *M1* are due to input from the cerebellum. It is also possible that repetitive stimulation of inputs to an area can result in short-term homeostatic regulation of network dynamics [72–74].

Cerebellar stimulation could also trigger activity-dependent synaptic plasticity [75, 76]. In general, brief periods of activity can trigger long-term potentiation and long-term depression, depending on the specific patterns of activation [72, 77]. Such activity can also increase or decrease the intrinsic excitability of presynaptic neurons [72], which might explain the observed diversity of single neuron plasticity following stimulation. It may also be possible, in future studies, to predict specific plasticity effects at the single neuron level by quantifying network connectivity with emerging computational methods [38].

Another possibility is that the observed changes in *M1* firing result from network plasticity in the cerebello-cortical system. Epidural DCS of the cerebellum has been shown to ‘focus’ corticomotor maps, by reducing the magnitude of corticomotor responses, and enhance afferent inhibition associated with peripheral stimuli [24]. Moreover, cerebellar stimulation can trigger changes in TMS, DCS and ICMS evoked MEPs [24, 36, 78–80]. The cerebellum projects mainly to cortical layers IV and V of *M1* via the ventrolateral thalamic nuclei [81]. These inputs can adjust *M1* circuitry in several ways, such as by modulating the efficacy of interconnections between *M1* neurons. Furthermore, the cerebellum receives numerous projections that predict and update sensory events through the interactions of mossy fibers and climbing fibers [82]. Thus, it is reasonable to suppose that large-scale network dynamics are modulated by cerebellar stimulation. It is also possible that observed changes in *M1* could be a result of plasticity at other sites.

### Increased neural synchrony during low frequency oscillations

It is important to note that the increases in neural synchrony and cell assembly activation strengths we observed occurred when the LFPs principally showed δ-waves, or LFOs [42, 83]. We didn’t observe a change in the LFP power of LFOs following stimulation. This suggests that changes in input to *M1* are not a main driver of the observed effects, as LFP is widely believed to be a measure of synaptic inputs [39, 44]. So what might be the broader physiological consequences of cerebellar stimulation-induced changes in correlated firing under LFO-like oscillations? It is known that this low frequency oscillatory activity commonly occurs during ketamine anesthesia [42, 83], but studies have shown that LFOs also occur at the spiking and LFP level in *M1* during reaching tasks [41, 42, 83, 84]. It is hypothesized that LFOs represent an intrinsic property of motor circuits that are involved in the production of fast and accurate movements. Stroke disrupts these movement related potentials in humans, which is highly correlated to motor impairments and recovery [42, 84]. Interestingly, our other recent work showed that parameters for the modulation of LFOs under anesthesia also generalized to awake skilled reaching [42]. Since, in this study, cerebellar stimulation enhanced neural synchrony while *M1* was predominantly characterized by LFOs, cerebellar stimulation might be particularly useful for modulating neural dynamics during cortical slow-wave oscillations. Therefore, future work could examine whether cerebellar stimulation similarly modulates movement-related spiking in intact and peri-infarct cortices in awake behaving animals. This might reveal one mechanism through which cerebellar stimulation could improve motor function in stroke patients.

## Conclusions

To summarize, a brief period of cerebellar stimulation resulted in long-lasting *M1* plasticity. We found that the firing rates and correlated firing changed significantly in response to stimulation, and that neural ensemble activation was boosted. Our findings here will help optimize cerebellar stimulation paradigms for those with motor disabilities post-stroke, or other movement disorders.

## Data Availability

The data that support the findings from this study are available from the lead contact upon reasonable request.

## Abbreviations

MEP: Motor evoked potentials
LFP: Local field potentials
M1: Motor cortex
PSD: Power spectral density
DCN: Deep cerebellar nuclei
tES: Transcranial electric stimulation
DCS: Direct current stimulation
MEA: Multielectrode array
SNR: Signal-to-noise ratio
*M*^+^: Positively-modulated neurons
*M*^*−*^: Negatively-modulated neurons
*NM*: Non-modulated neurons
CCH: Cross-correlation histogram
PCA: Principal component analyses
PC: Principal component
LFOs: Low-frequency oscillations
PBS: Phosphate buffered saline
DW: Distilled water
TMS: Transcranial magnetic stimulation
ICMS: Intracortical micro-stimulation
DBS: Deep brain stimulation
tDCS: Transcranial direct current stimulation
CBI: Cerebellar brain inhibition
DTC: Dentato-thalamo-cortical

## References

[1] Heck DH, De Zeeuw CI, Jaeger D, Khodakhah K, Person AL (2013). The neuronal code(s) of the cerebellum. J Neurosci.

[2] Allen GI, Tsukahara N (1974). Cerebrocerebellar communication systems. Physiol Rev.

[3] Thach WT (1970). Discharge of cerebellar neurons related to two maintained postures and two prompt movements. II. Purkinje cell output and input. J Neurophysiol.

[4] Fortier PA, Kalaska JF, Smith AM (1989). Cerebellar neuronal activity related to whole-arm reaching movements in the monkey. J Neurophysiol.

[5] Wetts R, Kalaska JF, Smith AM (1985). Cerebellar nuclear cell activity during antagonist cocontraction and reciprocal inhibition of forearm muscles. J Neurophysiol.

[6] van Kan PL, Houk JC, Gibson AR (1993). Output organization of intermediate cerebellum of the monkey. J Neurophysiol.

[7] Burton JE, Onoda N (1978). Dependence of the activity of interpositus and red nucleus neurons on sensory input data generated by movement. Brain Res.

[8] Burton JE, Onoda N (1977). Interpositus neuron discharge in relation to a voluntary movement. Brain Res.

[9] Chapman CE, Spidalieri G, Lamarre Y (1986). Activity of dentate neurons during arm movements triggered by visual, auditory, and somesthetic stimuli in the monkey. J Neurophysiol.

[10] Becker MI, Person AL (2019). Cerebellar control of reach kinematics for endpoint precision. Neuron.

[11] Kawato M, Wolpert D (1998). Internal models for motor control. Novartis Found Symp.

[12] Coltz JD, Johnson MT, Ebner TJ (1999). Cerebellar Purkinje cell simple spike discharge encodes movement velocity in primates during visuomotor arm tracking. J Neurosci.

[13] Fu QG, Flament D, Coltz JD, Ebner TJ (1997). Relationship of cerebellar Purkinje cell simple spike discharge to movement kinematics in the monkey. J Neurophysiol.

[14] Johnson MT, Coltz JD, Ebner TJ (1999). Encoding of target direction and speed during visual instruction and arm tracking in dorsal premotor and primary motor cortical neurons. Eur J Neurosci.

[15] Marple-Horvat DE, Stein JF (1987). Cerebellar neuronal activity related to arm movements in trained rhesus monkeys. J Physiol.

[16] Valle MS, Bosco G, Poppele R (2000). Information processing in the spinocerebellar system. NeuroReport.

[17] Bosco G, Poppele RE (2000). Reference frames for spinal proprioception: kinematics based or kinetics based?. J Neurophysiol.

[18] Heck DH, Thach WT, Keating JG (2007). On-beam synchrony in the cerebellum as the mechanism for the timing and coordination of movement. Proc Natl Acad Sci USA.

[19] Dayan E, Cohen LG (2011). Neuroplasticity subserving motor skill learning. Neuron.

[20] Shmuelof L, Krakauer JW (2011). Are we ready for a natural history of motor learning?. Neuron.

[21] Peters AJ, Chen SX, Komiyama T (2014). Emergence of reproducible spatiotemporal activity during motor learning. Nature.

[22] Rioult-Pedotti MS, Friedman D, Hess G, Donoghue JP (1998). Strengthening of horizontal cortical connections following skill learning. Nat Neurosci.

[23] Guo JZ (2015). Cortex commands the performance of skilled movement. Elife.

[24] Oulad Ben Taib N, Manto M (2013). Trains of epidural DC stimulation of the cerebellum tune corticomotor excitability. Neural Plast.

[25] Cooper IS (1981). Twenty-five years of experience with physiological neurosurgery. Neurosurgery.

[26] Rastogi A (2017). Modulation of cognitive cerebello-cerebral functional connectivity by lateral cerebellar continuous theta burst stimulation. Neuroimage.

[27] Naro A (2017). Effects of cerebellar transcranial alternating current stimulation on motor cortex excitability and motor function. Brain Struct Funct.

[28] Colnaghi S (2017). A role for NMDAR-dependent cerebellar plasticity in adaptive control of saccades in humans. Brain Stimul.

[29] Popa T (2013). Cerebellar rTMS stimulation may induce prolonged clinical benefits in essential tremor, and subjacent changes in functional connectivity: an open label trial. Brain Stimul.

[30] Bodranghien F, Oulad Ben Taib N, Van Maldergem L, Manto M (2017). A postural tremor highly responsive to transcranial cerebello-cerebral DCS in ARCA3. Front Neurol.

[31] Bonni S, Ponzo V, Caltagirone C, Koch G (2014). Cerebellar theta burst stimulation in stroke patients with ataxia. Funct Neurol.

[32] Wessel MJ, Hummel FC (2018). Non-invasive cerebellar stimulation: a promising approach for stroke recovery?. Cerebellum.

[33] Sebastian R (2016). Cerebellar tDCS: a novel approach to augment language treatment post-stroke. Front Hum Neurosci.

[34] Baker KB, Schuster D, Cooperrider J, Machado AG (2010). Deep brain stimulation of the lateral cerebellar nucleus produces frequency-specific alterations in motor evoked potentials in the rat in vivo. Exp Neurol.

[35] Machado AG, Baker KB, Schuster D, Butler RS, Rezai A (2009). Chronic electrical stimulation of the contralesional lateral cerebellar nucleus enhances recovery of motor function after cerebral ischemia in rats. Brain Res.

[36] Park HJ (2015). Modulation of cortical motor evoked potential after stroke during electrical stimulation of the lateral cerebellar nucleus. Brain Stimul.

[37] Asan AS, Lang EJ, Sahin M (2020). Entrainment of cerebellar purkinje cells with directional AC electric fields in anesthetized rats. Brain Stimul.

[38] Sadtler PT (2014). Neural constraints on learning. Nature.

[39] Buzsaki G (2010). Neural syntax: cell assemblies, synapsembles, and readers. Neuron.

[40] Gulati T, Guo L, Ramanathan DS, Bodepudi A, Ganguly K (2017). Neural reactivations during sleep determine network credit assignment. Nat Neurosci.

[41] Churchland MM (2012). Neural population dynamics during reaching. Nature.

[42] Ramanathan DS (2018). Low-frequency cortical activity is a neuromodulatory target that tracks recovery after stroke. Nat Med.

[43] Khanna P (2021). Low-frequency stimulation enhances ensemble co-firing and dexterity after stroke. Cell.

[44] Okun M (2015). Diverse coupling of neurons to populations in sensory cortex. Nature.

[45] Civillico EF, Contreras D (2012). Spatiotemporal properties of sensory responses in vivo are strongly dependent on network context. Front Syst Neurosci.

[46] Hishinuma AK, Gulati T, Burish MJ, Ganguly K (2019). Large-scale changes in cortical dynamics triggered by repetitive somatosensory electrical stimulation. J Neuroeng Rehabil.

[47] Asan AS, Sahin M (2019). Modulation of multiunit spike activity by transcranial AC stimulation (tACS) in the rat cerebellar cortex. Conf Proc IEEE Eng Med Biol Soc.

[48] Plautz EJ (2003). Post-infarct cortical plasticity and behavioral recovery using concurrent cortical stimulation and rehabilitative training: a feasibility study in primates. Neurol Res.

[49] Levy R (2008). Cortical stimulation for the rehabilitation of patients with hemiparetic stroke: a multicenter feasibility study of safety and efficacy. J Neurosurg.

[50] Gulati T (2015). Robust neuroprosthetic control from the stroke perilesional cortex. J Neurosci.

[51] Suner S, Fellows MR, Vargas-Irwin C, Nakata GK, Donoghue JP (2005). Reliability of signals from a chronically implanted, silicon-based electrode array in non-human primate primary motor cortex. IEEE Trans Neural Syst Rehabil Eng.

[52] Gulati T, Ramanathan DS, Wong CC, Ganguly K (2014). Reactivation of emergent task-related ensembles during slow-wave sleep after neuroprosthetic learning. Nat Neurosci.

[53] Peyrache A, Benchenane K, Khamassi M, Wiener SI, Battaglia FP (2010). Principal component analysis of ensemble recordings reveals cell assemblies at high temporal resolution. J Comput Neurosci.

[54] Lopes-dos-Santos V, Ribeiro S, Tort AB (2013). Detecting cell assemblies in large neuronal populations. J Neurosci Methods.

[55] Mitra P, Bokil H. Observed brain dynamics. xxii. 2008. 381 p.

[56] Aarts E, Verhage M, Veenvliet JV, Dolan CV, van der Sluis S (2014). A solution to dependency: using multilevel analysis to accommodate nested data. Nat Neurosci.

[57] Cooperrider J (2014). Chronic deep cerebellar stimulation promotes long-term potentiation, microstructural plasticity, and reorganization of perilesional cortical representation in a rodent model. J Neurosci.

[58] Shah AM (2017). Optogenetic neuronal stimulation of the lateral cerebellar nucleus promotes persistent functional recovery after stroke. Sci Rep.

[59] Grimaldi G (2016). Cerebellar transcranial direct current stimulation (ctDCS): a novel approach to understanding cerebellar function in health and disease. Neuroscientist.

[60] Ferrucci R, Bocci T, Cortese F, Ruggiero F, Priori A (2016). Cerebellar transcranial direct current stimulation in neurological disease. Cerebellum Ataxias.

[61] Galea JM, Jayaram G, Ajagbe L, Celnik P (2009). Modulation of cerebellar excitability by polarity-specific noninvasive direct current stimulation. J Neurosci.

[62] Doeltgen SH, Young J, Bradnam LV (2016). Anodal direct current stimulation of the cerebellum reduces cerebellar brain inhibition but does not influence afferent input from the hand or face in healthy adults. Cerebellum.

[63] Manto M, Gruol DL, Schmahmann JD, Koibuchi N, Rossi F (2012). Handbook of the cerebellum and cerebellar disorders.

[64] Nitsche MA (2003). Facilitation of implicit motor learning by weak transcranial direct current stimulation of the primary motor cortex in the human. J Cogn Neurosci.

[65] Nitsche MA, Paulus W (2001). Sustained excitability elevations induced by transcranial DC motor cortex stimulation in humans. Neurology.

[66] Butler AJ, Wolf SL (2007). Putting the brain on the map: use of transcranial magnetic stimulation to assess and induce cortical plasticity of upper-extremity movement. Phys Ther.

[67] Di Lazzaro V (1994). Cerebro-cerebellar interactions in man: neurophysiological studies in patients with focal cerebellar lesions. Electroencephalogr Clin Neurophysiol.

[68] Werhahn KJ, Taylor J, Ridding M, Meyer BU, Rothwell JC (1996). Effect of transcranial magnetic stimulation over the cerebellum on the excitability of human motor cortex. Electroencephalogr Clin Neurophysiol.

[69] Park HJ (2015). Modulation of cortical motor evoked potential after stroke during electrical stimulation of the lateral cerebellar nucleus. Brain Stimul.

[70] Machado A, Baker KB (2012). Upside down crossed cerebellar diaschisis: proposing chronic stimulation of the dentatothalamocortical pathway for post-stroke motor recovery. Front Integr Neurosci.

[71] Chan HH (2018). Lateral cerebellar nucleus stimulation has selective effects on glutamatergic and GABAergic perilesional neurogenesis after cortical ischemia in the rodent model. Neurosurgery.

[72] Ganguly K, Poo MM (2013). Activity-dependent neural plasticity from bench to bedside. Neuron.

[73] Feldman DE (2009). Synaptic mechanisms for plasticity in neocortex. Annu Rev Neurosci.

[74] Castro-Alamancos MA, Donoghue JP, Connors BW (1995). Different forms of synaptic plasticity in somatosensory and motor areas of the neocortex. J Neurosci.

[75] Francis JT, Song W (2011). Neuroplasticity of the sensorimotor cortex during learning. Neural Plast.

[76] Fritsch B (2010). Direct current stimulation promotes BDNF-dependent synaptic plasticity: potential implications for motor learning. Neuron.

[77] Markram H, Lubke J, Frotscher M, Sakmann B (1997). Regulation of synaptic efficacy by coincidence of postsynaptic APs and EPSPs. Science.

[78] Iwata NK, Ugawa Y (2005). The effects of cerebellar stimulation on the motor cortical excitability in neurological disorders: a review. Cerebellum.

[79] Ugawa Y, Uesaka Y, Terao Y, Hanajima R, Kanazawa I (1995). Magnetic stimulation over the cerebellum in humans. Ann Neurol.

[80] Oliveri M, Koch G, Torriero S, Caltagirone C (2005). Increased facilitation of the primary motor cortex following 1 Hz repetitive transcranial magnetic stimulation of the contralateral cerebellum in normal humans. Neurosci Lett.

[81] Sanes JN, Donoghue JP (2000). Plasticity and primary motor cortex. Annu Rev Neurosci.

[82] Nowak DA, Topka H, Timmann D, Boecker H, Hermsdorfer J (2007). The role of the cerebellum for predictive control of grasping. Cerebellum.

[83] Hall TM, de Carvalho F, Jackson A (2014). A common structure underlies low-frequency cortical dynamics in movement, sleep, and sedation. Neuron.

[84] Yilmaz O, Cho W, Braun C, Birbaumer N, Ramos-Murguialday A (2013). Movement related cortical potentials in severe chronic stroke. Conf Proc IEEE Eng Med Biol Soc.

